# Correction: The ribosomal RPL10 R98S mutation drives IRES-dependent BCL-2 translation in T-ALL

**DOI:** 10.1038/s41375-019-0424-x

**Published:** 2019-03-08

**Authors:** Kim R. Kampen, Sergey O. Sulima, Benno Verbelen, Tiziana Girardi, Stijn Vereecke, Laura Fancello, Gianmarco Rinaldi, Jelle Verbeeck, Joyce Op de Beeck, Anne Uyttebroeck, Jules P. P. Meijerink, Anthony V. Moorman, Christine J. Harrison, Pieter Spincemaille, Jan Cools, David Cassiman, Sarah-Maria Fendt, Pieter Vermeersch, Kim De Keersmaecker

**Affiliations:** 1 Department of Oncology, Laboratory for Disease Mechanisms in Cancer, KU Leuven and Leuven Cancer Institute (LKI), Leuven, Belgium; 2 Laboratory of Cellular Metabolism and Metabolic Regulation, Center for Cancer Biology, VIB, Leuven, Belgium; 3 Department of Oncology, Laboratory of Cellular Metabolism and Metabolic Regulation, KU Leuven and Leuven Cancer Institute (LKI), Leuven, Belgium; 4 Department of Pediatric Oncology & Hematology, University Hospitals Leuven, Leuven, Belgium; 5 Princess Máxima Center for Pediatric Oncology, Utrecht, The Netherlands; 6 Leukaemia Research Cytogenetics Group, Northern Institute for Cancer Research, Newcastle University, Newcastle-upon-Tyne, UK; 7 Department of Gastroenterology-Hepatology and Metabolic Center, University Hospitals Leuven, Leuven, Belgium; 8 Laboratory of Molecular Biology of Leukemia, Center for Human Genetics, KU Leuven and Leuven Cancer Institute (LKI), Leuven, Belgium; 9 Laboratory of Molecular Biology of Leukemia, Center for Cancer Biology, VIB, Leuven, Belgium; 10 Department of Laboratory Medicine, University Hospitals Leuven, Leuven, Belgium

**Correction to:** **Leukemia** **33**, 319–332 (2019);

10.1038/s41375-018-0176-z;

published online 21 February 2019.

Following the publication of this article, the authors noted that Dr Laura Fancello was not listed among the authors. The corrected author list is given below. Additionally, the following was not included in the author contribution statement: ‘L.F. analyzed RNA sequencing data’.

The authors wish to apologise for any inconvenience caused.

Kim R. Kampen^1^, Sergey O. Sulima^1^, Benno Verbelen^1^, Tiziana Girardi^1^, Stijn Vereecke^1^, Laura Fancello^1^, Gianmarco Rinaldi^2,3^, Jelle Verbeeck^1^, Joyce Op de Beeck^1^, Anne Uyttebroeck^4^, Jules P. P. Meijerink^5^, Anthony V. Moorman^6^, Christine J. Harrison^6^, Pieter Spincemaille^7^, Jan Cools^8,9^, David Cassiman^7^, Sarah-Maria Fendt^2,3^, Pieter Vermeersch^10^, Kim De Keersmaecker^1^

